# Exploring the Association between Vitamin D and Changes in Cognitive Function in Chilean Older Adults: Evidence from the ALEXANDROS Cohort Study

**DOI:** 10.3390/jpm12071078

**Published:** 2022-06-30

**Authors:** Carlos Márquez, Bárbara Angel, Lydia Lera, Rebecca Bendayan, Hugo Sánchez, Cecilia Albala

**Affiliations:** 1 Public Health Nutrition Unit, Institute of Nutrition and Food Technology, University of Chile, Santiago 7830490, Chile; cmarquez@inta.uchile.cl (C.M.); llera@inta.uchile.cl (L.L.); hugo.sanchez@hurtadohosp.cl (H.S.); 2 Latin Division, Keiser University eCampus, Fort Lauderdale, FL 33409, USA; 3 Department of Biostatistics and Health Informatics, Institute of Psychiatry, Psychology and Neuroscience, King’s College London, London SE5 8AF, UK; rebecca.bendayan@kcl.ac.uk; 4 NIHR Biomedical Research Centre at South London and Maudsley, NHS Foundation Trust and King’s College London, London SE5 8AF, UK

**Keywords:** aging, vitamin D, cognitive function, mild cognitive impairment

## Abstract

Background: The increasing aging of the population with the consequent increase of age-associated cognitive disorders pose the challenge of controlling its preventable risk factors, among which vitamin D deficit is a putative factor. Thus, our objective is to explore the association between vitamin D and cognitive performance in a cohort study of community-dwelling Chilean older people. Material and Methods: Cohort study of 955 (69.7% female), community-dwelling older Chileans free of cognitive impairment from the Alexandros cohorts, with 25(OH)D measurement at baseline. Cognitive Function was evaluated with the Mini Mental State Examination (MMSE) short-form questionnaire. Plasma levels of 25(OH)D were classified as Normal > 30 ng/mL Insufficiency 20–29 ng/mL, Deficiency 20–12 ng/mL and Severe Deficiency < 12 ng/mL. Penalized regressions models were made to assess associations. Results: Mean age of the sample was 66.6 + 4.5 years, with 8.5 + 4.7 years of education. After a mean follow-up of 9.6 years, 54 new cases of Mild Cognitive Impairment (MCI)were identified (Incidence density rate = 5.9 per 1000 person/years). Mean vitamin D plasma levels were lower in people with MCI than in the normal cognitive ones (23.0 + 12.75 vs. 28.35 + 15.17 ng/mL, *p* < 0.01). In the fully adjusted model only severe deficiency of vitamin D was associated with MCI (RR = 2.33; 95% CI: (1.03–5.26). Conclusions: In this longitudinal study, our results confirm that low Vitamin D is a risk factor for MCI, and that people with severe deficiency have more than double the risk of MCI people with normal Vitamin D levels. Considering the high frequency of vitamin D deficiency in older people, and its preventability, these results are very valuable for future public health programmes.

## 1. Introduction

The accelerated process of demographic and epidemiological transition undergone by Latin America (LA) has caused a rapid aging of the population. Chile is the country in LA that has most increased its life expectancy at birth (LEB). Between 1970 and 2019, LEB increased from 60.5 to 78.1 years in men and from 66.8 to 83.2 years for women [1]. Besides, the 65 years and older population increased from 4.9% in 1980 to 12.2% in 2020 [2].

Associated with the aging process, there has been an increase in the frequency of chronic and neurodegenerative diseases [3]. Among the latter, the most frequent is dementia, an important public health problem recognized by the WHO as a public health priority [4], considering that cognitive decline and dementia poses a significant burden for societies and health care systems, especially in low and middle-income countries [5]. The Global Burden of Diseases (GBD) 2019 study estimates more than 57 million people with dementia, forecasting an increase of 166% in 2050 to more than 152 million [6].

Alzheimer’s disease and other dementias account for 1.2% of total disability in America, ranging from 0.4% in Haiti to 1.9% in Canada. They reflect a highly significant trend, which correlates a higher Gross Domestic Product (GDP) with a higher percentage of total disability attributable to dementias. This is an expression of demographic change in the age structure, where high survival and disability due to disorders in older adults accompany economic development [7]. However, the rapid ageing of the population in countries of low and medium-low income allows to forecast an important increase of dementia [6] and disability attributable to dementia in these countries.

Studies of cognitive function in Latin America have focused particularly on the diagnosis of dementia and its impact on health systems [8,9,10,11]. Given the fact that the proportion of people with dementia in Latin America is estimated to increase by 2–3, with higher increase in countries of lower income [6] there is an urgent need to further explore cognitive changes and its correlates in ageing population.

Diseases associated with cognitive decline are probably the most feared and devastating health problems that affect older people and their families [12]. These diseases are modulated by multiple factors: Genetic, vascular, lifestyle, and contextual (Education) and are some of the main causes of disability and dependence worldwide [13]. According to GBD Study, 40% of dementia cases could be prevented by modifying preventable risk factors through the life course [14]. Besides the well-recognized socioeconomic factors such as low education and isolation, those related with lifestyles, mainly nutrition and physical activity and chronic diseases such as diabetes, hypertension, obesity and depression have emerged as the most important preventable risk factors.

The lifestyle-cognition hypothesis holds that maintaining an active lifestyle and engaging in certain activities during one’s life may help prevent age-associated cognitive decline [15]. Cognitive function may benefit from nutrition [16] and dietary patterns may be important in the prevention of cognitive decline [17]. The studies of diet-related risk of cognitive decline and dementia suggests that nutrients such as vitamins and trace-elements can prevent cognitive decline [18]. It has been observed that subjects with low blood levels of vitamins D, C, B2, B9 and B12 have shown worse performance on cognitive tests [19]. Several cross-sectional studies have shown association of Vitamin D deficiency with cognitive impairment [20,21] although meta-analysis of longitudinal studies are not conclusive [22].

Studying the impact of vitamin D deficiency on cognition in an aging population is especially important, considering that nearly one in two older adults and 70–90% of adults with cognitive difficulties have hypovitaminosis D [23]. Some studies have shown that hypovitaminosis D increases the risk of cognitive decline and dementia in older adults [24] but a review on the association between vitamin D and cognitive function in the European, Asian and North American population was not conclusive [25].

In Chile, there is a high prevalence of Vitamin D deficiency in the population, higher in this age group [26]. According to the last National Health Survey, there is a 60% of Vitamin D deficit prevalence in people of aged 65 and older, even when there is a public health program for complementary feeding for the elderly whose objective is to contribute to preventing and treating micro-nutrients deficiency in this group [27]. Despite this high prevalence of Vitamin D deficiency, no previous studies in Chile have analyzed its association with cognitive decline.

The background of the rapid and growing process of aging of the Chilean population, the high prevalence of Vitamin D deficiency in older people and the associations between vitamin D deficiency levels and cognitive function decline, make us think that this study could be a contribution to public health policies in Chile, since there are no longitudinal studies that establish an association between vitamin D deficiency and mild cognitive impairment in our population, the ALEXANDROS study is one of the few cohorts that exist in Chile and the only one that evaluates the effect of nutritional status in older people Longitudinally, the methodology of this cohort study allows us to define risk factors in older people. Considering these antecedents, our general objective is to observe cognitive performance and its variations, exploring the association between vitamin D and cognitive performance in a cohort study of community-dwelling Chilean older people.

## 2. Materials and Methods

### 2.1. Setting and Sample

Follow-up study done in the context of Alexandros (Active Life Expectancy, Aging and Disorders Related to Obesity Study) after a mean follow up of 9078 person/years (mean 9.59 ± 3.5, min 5.0 max 15.0), a cohort study of community-dwelling people 60 years and older living in Santiago, Chile, previously described [28]. Briefly Alexandros is a longitudinal study of people ≥ 60 years that includes three cohorts of different demographic origin and socioeconomic status: (a) 1173 Subjects born before 1940 recruited in the frame of The SABE study [29] (in 1999–2000 through a probabilistic sampling), (b) 950 people born between 1940–1948 recruited between 2005 and 2008, randomly selected from 28 Public Primary healthcare center registries and (c) 266 people of high socio-economic status, born before 1949, randomly selected from the private health insurance system registries and recruited in 2007. After approval by the Institutional Scientific Ethics Review Board of the Institute of Nutrition and Food Technology (INTA) of the University of Chile (Acta n°23, 2012, Fondecyt n°1130947), and informed consent signature, biomedical, sociodemographic, anthropometric, and functional evaluations were performed, and a fasting blood sample was obtained. The Inclusion criteria for this study was individuals with measurements of vitamin D Levels at baseline and Cognitive Function (MMSE short form) evaluation. The exclusion criteria were having a MMSE short form score < 13 The sample consisted of 955 participants (69.7% women) 60 years and older followed since its recruitment.

### 2.2. Variables

Cognitive Function Evaluated through a validated neuropsychological screening test in Chilean population: Mini Mental State Examination (MMSE) short form and defining normal cognition as MMSE greater or equal than 13 Points, MCI as MMSE less than 13 [30]. Vitamin D. The measurement of vitamin D was done in Spring/Summertime for all participants. Plasma levels of 25OH vitamin D (25OHD), were measured through the radioimmunoassay technique (RIA). Normal status is defined as a 25(OH)D ≥ 30 ng/mL (75 nmol/L), Insufficiency as 20–29 ng/mL and Deficiency as a 25(OH)D level 12–19 ng/mL and severe deficiency as <12 ng/mL [31,32].

### 2.3. Covariates

Socio-demographic variables included were age, sex, Years of education and socioeconomic position (SEP). The lifestyles characteristics included were alcohol consumption, level of physical activity and smoking, all of them self-reported. Comorbidities where assessed through self-report of previous diagnostic from a questionnaire of pathologies including: high blood pressure, diabetes, heart disease, lung disease, arthritis, stroke and others. Depression was assessed with the Geriatric Depression Scale (GDS-15) questionnaire [33] using the score ≥ 5 for depression diagnostic. Number of diseases, Multimorbidity (2 or more diseases). Weight and height variables were used to calculate the body mass index (BMI: Weight/Height^2^ = Kg/m^2^) and determine nutritional status using the BMI categories according to the WHO Cut-off points.

### 2.4. Statistical Analysis

Preliminary and exploratory analyses were performed to check the normality of the distribution of the MMSE short form, Vitamin D, and anthropometric variables. For this, the Kolmogorov-Smirnov test and some graphic evidence were used. Descriptive analyses of continuous variables were performed and mean, standard deviations are provided. Rank and percentile distribution was provided for categorical variables. Chi-square and Pearson correlation tests were performed to run exploratory analyses on relevant variables at baseline and follow up. To examine whether there was an association between basal vitamin D levels and cognitive function at follow-up, a series of Firth’s penalized logistic regression models were performed. All the regression models analyses were performed on independent models; the first was performed unadjusted, the second model was adjusted for socio-demographics age, sex and education (Adjusted), and the third model was adjusted for health status measured by chronic diseases, number of chronic disease and BMI (Full adjusted). All statistical analyses were performed using STATA 15 (StataCorp. 2017. Stata Statistical Software: Release 15. College Station, TX, USA: StataCorp LP).

## 3. Results

### 3.1. Sample Description

The sample was composed of 955 subjects (69.7% women), 60 years and older with a mean age of 66.6 ± 4.52 years, similar in men and women. The general characteristics of the sample are described in Table 1. The mean years of education were 8.5 ± 4.4 with no differences between genders. A high proportion of the sample was sedentary (71.1%). On average the participants have 1.2 ± 1.01 diseases, 17.8% have diabetes, 57.8% have high blood pressure and 37.1% have depression. The mean BMI was 28.9 ± 4.86 Kg/m^2^, higher in women than men. Alcohol consumption was higher in men than women (31.4 vs. 14.3%) and smoking, although less frequent, was also higher in men than women (9.7 vs. 5.4%). Mean score of MMSE was 17, similar in both genders and mean Vitamin D plasma levels were higher in men than in women (29.61 ± 15.3 vs. 27.4 ± 14.9, *p* < 0.035)

### 3.2. Association between Health Factors and Vitamin D

Table 2 shows the cognitive status, nutritional state, depression, and self-reported diabetes, according to plasma Vitamin D. MCI proportion was higher the lower the 25OHD status category (*p* = 0.005). The difference in the frequency of MCI is given by Vitamin D severe deficiency: 11.0% of subjects in this category had MCI compared with 7.8% in the Deficiency group and 4.0% in the Normal ones. No differences were observed according to BMI categories, depression, or diabetes. Mean plasma levels of 25OHD according to cognitive status (not shown), were higher in subjects with normal cognition when compared with subjects with MCI (28.35 ng/mL vs. 23.05 ng/mL, *p* < 0.01).

### 3.3. Association between Vitamin D and Cognitive Function

After a mean follow up of 9078 person/years (mean 9.59 ± 3.5, min 5.0 max 15.0), 54 new cases of MCI were identified, yielding an incidence density of = 0.59 per 100 person/years. The penalized regression models exploring the association between 25(OH)D basal levels and cognitive status at follow-up are shown in Table 3. In the fully adjusted model including sociodemographic variables, nutritional status and multimorbidity, severe deficiency more than doubles the risk of MCI (RR: 2.33 CI95%: 1.06–5.12), compared to the normal 25(OH)D group, with the risk being 79% higher for women than men and 15% lower with each year of education attained.

## 4. Discussion

In this longitudinal study, after a mean follow-up of 9078 person/years (mean 9.59 ± 3.5, min 5.0 max 15.0), The participants present a high prevalence of sedentarism (71.1%), high blood pressure (57.8%) and depression (37.1%), the average body mass index was 28.88 kg/m^2^ with a prevalence of obesity of 46%, in the follow-up study a prevalence of 6.7% of MCI was detected with significantly lower plasma vitamin D levels compared to participants classified as cognitively normal (23.05 ng/mL vs. 28.35 ng/mL, *p* < 0.01). In addition, we have found that cognitive performance is associated with vitamin D levels at baseline. Like most studies, we have used MMSE for the diagnosis of cognitive impairment and dementia. Our findings shows that vitamin D deficiency is associated with the development of mild cognitive impairment over time, thus observing a higher prevalence of mild cognitive impairment in people with severe vitamin D deficiency versus normal vitamin D levels (11% vs. 4.01% *p* < 0.005).

Many cross-sectional studies have shown similar results. A study conducted in Mexico City with 208 subjects with a mean age of 79 and an average educational level of 6.7y found Vitamin D deficiency in 64% of the subjects with dementia, 59% in the MCI group and 13% in the subjects with normal cognition [20]. The strong association of MCI and Dementia with Vitamin D deficiency persisted after the multivariate logistic regression analysis.

Another cross-sectional study conducted at the Fukuoka University Hospital including 230 participants assigned into three groups (Healthy, MCI and Alzheimer’s disease), concluded that that the serum concentration of 25(OH)D3 is predictive of severe AD and contributes to MMSE variability, and so could be a useful biomarker in the prediction and diagnosis of mild cognitive impairment. [21]. In a study conducted at the Shanghai Jiaotong University Hospital, with 299 participants aged 65 years or older, where cognitive function was also evaluated with MMSE, Vitamin D status was classified into three groups: A (<10.0 ng/mL), B (10.0–19.9 ng/mL) and C (≥20.0 ng/mL). Among the results of the study, we can highlight that the A group had a greater number of patients with cognitive impairment when compared with groups B and C [34].

In our study the fully adjusted model shows that in people with 25(OH)D severe deficiency, the risk of MCI is more than twice (RR = 2.3; 95% CI: 1.03–5.26) the risk of people with normal 25(OH)D levels.

Reviews of longitudinal studies have shown evidence that as serum vitamin D decreases, cognitive function also decreases and that the severe vitamin D (<25 nmol/L) deficiency is a significant risk for the decline of global cognition measured with MMSE [35], although results revealed a significant, but weaker effect of this association compared with those seen in cross-sectional studies. This is likely attributed to age and gender differences, as well as follow-up durations between different studies [36].

Other longitudinal studies have shown that low vitamin D concentrations at baseline were associated with higher risk of incident global cognitive decline [37]. In the Tromsø Study, the MMSE score was positively associated with serum vitamin D levels in people 65y and older in which vitamin D predicted cognitive function 7–13 years later [38].

Some studies have more specific results focusing on the cognition domains affected by Vitamin D deficiency. In the Women’s Healthy Ageing Project the status of vitamin D was associated with executive function [39]. The prospective study of Annweiler [40] showed that baseline hypovitaminosis D precedes cognitive decline specifically in processing speed evaluated using Trail Making Test. In a population-based longitudinal study of 1058 adults followed up over 12 years, moderately low Vitamin D was associated with poorer performance on multiple domains of cognitive function, although Low Vitamin D did not predict 12-year cognitive decline [22].

On the other hand, a meta-analysis using data from 17 studies with 172,349 participants has not found evidence for a causal association between vitamin D and cognitive function [25].

These different findings may be due to various factors. The proportions of vitamin D deficiency differ between studies, as well as how to assess cognition, median age, total follow-up time and type of study.

The findings of our study have important repercussions considering the high prevalence of vitamin D deficiency in this population. Currently, the Chilean population, as well as the worldwide population, presents an important deficit of vitamin D not only in the older adult population but also in other age groups, as is the case studied on school children from Punta Arenas-the southernmost city of the country, located at 53° degrees South-who have a high prevalence of vitamin D deficiency, most in the range of severe deficiency [41]. The national survey of chronic diseases carried out in 2016/2017 found that about half of the population of older people have Vitamin D levels under 20 ng/mL with higher prevalence of deficit in the southern part of the country with high latitudes.

A study carried out in Santiago de Chile in 2013 in older people aged 60 to 98 years found a prevalence of 36.5% deficit in men and 40.8% in women [42]. When assessing this deficit in different regions of the country, there was an effect related to latitude where older people live in Chile, finding a deficit of 8.4% in the north of the country, 37.5% in the central area and 66.8% in the south region [43].

Among the strengths of this Study is that it is the first longitudinal study in Chile exploring the association between cognitive performance and vitamin D, in a sample of about 1000 subjects. A limitation of the study is the important disparities in educational levels according to socioeconomic status that influence MMSE score, but this sample is mainly of people of low-medium socioeconomic status and the MMSE has been validated in Chile as a screening tool for cognitive status [44].

Vitamin D is a relevant nutrient in older people because besides its multiple functions, the changes occurring in old age favor this deficit because of absorption problems, storage and nutrient utilization, difficulty accessing food and feeding, high frequency of polypharmacy with probable drug interactions, and finally, a possible increase in the needs and excretion of micronutrients [45]. Besides, with advancing age, the capacity of the skin to produce vitamin D_3_ decreases (irrespective of the season) [46].

A point not addressed in our study is that there is solid evidence to support that obesity or overweight may cause cognitive comorbidity like depression and anxiety [47]. Considering the high prevalence of depression and obesity detected in our study, it is necessary to question in the future the relationship and the possible interaction between them, in addition to the effect they may have on the mental health of the elderly.

This study is relevant from a public health perspective considering the rapid aging of the population with the consequent increase of cognitive disorders and the high prevalence of Vitamin D deficiency in older Chileans. The latter could be considered a modifiable risk factor for cognitive performance decline, thus pointing out the need of public health policies and programs to face this important public health problem.

## Figures and Tables

**Table 1:** General characteristics of the sample by sex.

Characteristics	Men *n* = 289	Women *n* = 666	Total *n* = 955	Statistics Test *p* Value
Age (Years)	66.59 ± 4.57	66.58 ± 4.50	66.58 ± 4.52	<0.98 *
MMSE SF (points) Median, mean ± SD	17 17.02 ± 1.63	17 16.69 ± 1.71	17 16.79 ± 1.69	<0.37 ***
Vitamin D ng/mL mean SD	29.61 ± 15.33	27.37 ± 14.94	28.05 ± 15.08	<0.035 *
Years of Education mean ± SD	8.87 ± 4.89	8.29 ± 4.61	8.47 ± 4.70	<0.09 *
Number of chronic diseases mean ± SD	1.15 ± 0.96	1.27 ± 1.03	1.23 ± 1.01	<0.11 *
Diabetes *n* (%)	44 (16.42)	110 (18.43)	154 (17.80)	<0.46 **
High Blood Pressure *n* (%)	170 (58.82)	374 (56.24)	544 (57.02)	<0.45 **
Depression *n* (%)	20 (16.26)	122 (46.92)	142 (37.08)	<0.0001
BMI (Kg/m^2^) mean ± SD	27.95 ± 4.30	29.29 ± 5.04	28.88 ± 4.86	0.0002 *
Smoking *n* (%)	14 (5.36)	57 (9.68)	71 (8.35)	<0.036 **
Drinking *n* (%)	81 (31.40)	84 (14.26)	165 (19.48)	<0.001 **
Sedentary *n* (%)	191 (73.46)	409 (70.03)	600 (71.09)	<0.31 **

MMSE SF: Mini mental State Examination Short Form, BMI: Body Mass Index, SD: Standard deviation, * T-Students paired mean, ** Pearson chi^2^ test, *** Sd test mean.

**Table 2:** Vitamin D status according to cognitive status, nutritional state, depression and diabetes.

	Vitamin D Categories				
Characteristics	Normal (>20 ng/mL) *n* = 623	Deficiency (20–12 ng/mL) *n* = 232	Severe Deficiency (<12 ng/mL) *n* = 100	Total *n* = 955	Statistics Test *p* Value
Cognitive status *n* (%)	-	-	-	-	-
Normal	598 (95.99)	214 (92.24)	89 (89.0)	901 (94.34)	0.005 *
Mild Cognitive Impairment	25 (4.01)	18 (7.76)	11 (11.0)	54 (6.66)	-
Nutritional Status *n* (%)	-	-	-	-	0.595 *
Normal	127 (20.39)	42 (18.10)	18 (18.0)	187 (19.58)	
Undernutrition	1 (0.16)	0	0	1 (0.10)	
Overweight	298 (47.83)	94 (40.52)	48 (48.0)	440 (46.01)	
Obesity	197 (31.62)	96 (41.38)	34 (34.0)	327 (34.24)	
Depression (GDS-15) *n* (%)	-	-	-	-	-
Yes	56 (8.99)	14 (6.03)	10 (10.0)	80 (8.38)	0.436 *
No	567 (91.01)	218 (93.97)	90 (90.0)	875 (91.62)	-
Diabetes *n* (%)	-	-	-	-	-
Yes	101 (16.21)	42 (18.10)	22 (22.0)	165 (17.28)	0.339 *
No	522 (83.79)	190 (81.90)	78 (78.0)	790 (82.72)	

GDS: Geriatric Depression Scale, * Pearson chi^2^ test.

**Table 3:** Penalized logistic models for Mild cognitive Impairment risk According to baseline vitamin D status.

Variables	Unadjusted RR (95% CI)	Adjusted RR (95% CI)	Full Adjusted RR (95% CI)
Baseline Vitamin D	-	-	-
Deficiency (12–19 ng/mL)	1.73 (0.86–3.48) *p* 0.123	1.34 (0.64–2.81) *p* 0.427	1.25 (0.64–2.85) *p* 0.420
Severe deficiency (<12 ng/mL)	3.31 (1.52–7.17) *p* 0.002	2.36 (1.05–5.31) *p* 0.038	2.34 (1.03–5.312) *p* 0.042
Age (Years)	-	1.13 (1.07–1.21) *p* 0.0001	1.14 (1.07–1.21) *p* 0.0001
Sex (Women)	-	1.93 (0.85–4.37) *p* 0.114	1.71 (0.75–3.92) *p* 0.20
Years of Education	-	0.84 (0.78–0.92) *p* 0.0001	0.85 (0.78–0.93) *p* 0.0001
Nutritional Status	-	-	-
Overweight (25–29.9 Kg/m^2^)	-	-	0.80 (0.32–1.99) *p* 0.61
Obesity (≥30 Kg/m^2^)	-	-	0.93 (0.36–2.49) *p* 0.94
Depression	-	-	2.24 (1.17–4.29) *p* 0.01
Diabetes	-	-	1.22 (0.49–3.06) *p* 0.67
High blood pressure	-	-	0.86 (0.39–1.89) *p* 0.71
Multimorbidity	-	-	0.90 (0.40–1.76) *p* 0.64

RR: Relative Risk, 95% CI: 95% confidence intervals.
